# N6-methyladenosine-modified SRD5A3, identified by IGF2BP3, sustains cisplatin resistance in bladder cancer

**DOI:** 10.1007/s13577-024-01136-0

**Published:** 2024-12-16

**Authors:** Kai Liao, Jing Li, Caixian He, Jiyong Peng

**Affiliations:** 1 Department of Radiotherapy, Guangzhou Institute of Cancer Research, The Affiliated Cancer Hospital, Guangzhou Medical University, No. 78 Hengzhigang, Yuexiu District, Guangzhou, 510095 China; 2 Department of Urinary Surgery, Guangzhou Institute of Cancer Research, The Affiliated Cancer Hospital, Guangzhou Medical University, Guangzhou, 510095 China

**Keywords:** Bladder cancer, SRD5A3, IGF2BP3, N6-methyladenosine, Cisplatin resistance

## Abstract

Resistance to cisplatin-based chemotherapy limits the clinical benefit to some bladder cancer patients, and understanding the epigenetic regulation mechanism of cisplatin (CDDP) resistance in bladder cancer from the perspective of N6-methyladenosine (m6A) modification may optimize CDDP-based treatments. The study identified SRD5A3 as an oncogene for bladder cancer and stabilized by a m6A reader, IGF2BP3, to sustain CDDP resistance. Our results revealed that the expression of SRD5A3 was elevated in human bladder cancer tissues and cell lines, and this elevation was more evident in CDDP-resistant T24 and 5637 cells. Results of CCK-8 assay, colony formation assay, EdU staining, and flow cytometric analysis revealed that SRD5A3 knockdown and IGF2BP3 knockdown reduced cell proliferation and prevented chemoresistance in CDDP-resistant T24 and 5637 cells. Results of methylated RNA immunoprecipitation-PCR, RNA immunoprecipitation assay, and luciferase reporter assay showed IGF2BP3 recognized the SRD5A3 m6A modification and stabilized its mRNA. Nude mice implanted subcutaneously with CDDP-resistant T24 cells were injected intraperitoneally with CDDP (2 mg/kg) every 3 days for 35 days and the results demonstrated that SRD5A3 knockdown and IGF2BP3 knockdown effectively inhibited the tumor growth in subcutaneous implantation model. Collectively, the study unveils that IGF2BP3-mediated SRD5A3 m6A modification facilitates bladder cancer progression and induces CDDP resistance, providing rational therapeutic targets for bladder cancer patients.

## Introduction

Bladder cancer remains one of the most frequently occurring cancers globally and contributes to considerable morbidity and mortality, thus becoming a rising burden for health-care systems [1, 2]. Among bladder cancer patients, the overwhelming majority (nearly 90%) are urothelial carcinomas and 70–85% of urothelial carcinomas are recognized as non-muscle-invasive bladder cancer (NMIBC) [3]. Tobacco smoking and exposure to suspected carcinogens, such as aromatic amines, in workplaces have been deemed as the most substantial risk factors of bladder cancer [4]. As a heterogeneous disease, bladder cancer exhibits a variable natural history, and its progression brings a heavy treatment burden on patients [5, 6]. However, a recurrence rate after initial transurethral resection ranges from 50 to 80%, and 10–25% of these recurrences upstaging to muscle-invading tumor [7]. This clinical issue raises a need for a comprehensive knowledge of tumor genesis pathways in bladder cancer [8, 9]. Cisplatin-based chemotherapy remains a dominated systemic therapy to improve survival outcomes for patients with muscle-invasive bladder cancer (MIBC) [10]. The overall response rate to cisplatin-based chemotherapy is 40–50% in MIBC patients, and the 5 years survival rate of non-responders to cisplatin-based chemotherapy remains 30–40%, creating a critical need to identify those will really benefit from cisplatin-based chemotherapy and to prevent intrinsic or acquired resistance to cisplatin (CDDP) [11]. Treatment failure due to marked resistance to CDDP in tumor cells is a cause to worry, while the natural history and genomic landscape that orchestrates CDDP resistance remain to be elucidated [12].

In recent years, epigenetic modifications, such as DNA methylation and m6A modification, have gathered much attention due to its implications in intrinsic and acquired CDDP resistance in bladder cancer cells [13]. N6-methyladenosine (m6A) modification, a post-transcriptional RNA modification, is reversible and can be dynamically controlled by three categories of enzymes: writers, erasers, and readers, which is presumed to be a crucial mechanism in cancer development and treatment failure [14]. Insulin-like growth factor-II mRNA-binding protein (IGF2BP) family (IGF2BP1-3) belong to m6A reader proteins that have ability to enhance mRNA stability and translation [15]. Among this family, although IGF2BP3 is newly reported, it has been extensively studied to increase the mRNA stability and translation of oncogenes thus promoting tumorigenesis in several human cancers, such as pancreatic cancer [16], kidney cancer [17], gastric cancer [18], lung cancer [19], breast cancer [20], and hepatocellular carcinoma [21]. Of note, an upregulated IGF2BP3 may fuel disease progression, indicating an unfavorable prognostic factor in bladder cancer [22]. Steroid 5 alpha-reductase 3 (SRD5A3) is a member of the steroid 5-alpha-reductase family that catalyze testosterone conversion into natural androgen 5 alpha-dihydrotestosterone (DHT) and thus fine-tune the generation of steroid hormones and promote male sexual development [23, 24]. As an isoenzyme of SRD5A1 and SRD5A2, SRD5A3 has been discovered lately and it is demonstrated as an oncogene and confers a poor prognosis in several human cancers, such as breast cancer [25] and hepatocellular carcinoma [26]. Silencing SRD5A3 was demonstrated to decrease DHT production in prostate cancer cells and reduce cell viability, indicating its tumor-promoting activity in prostate cancer [27]. However, the role of SRD5A3 during bladder tumorigenesis and subsequent CDDP resistance has not yet been depictured. In this study, we sought to demonstrate the oncogenic role of SRD5A3 and its interplay with IGF2BP3 via a m6A-dependent manner in bladder cancer and CDDP resistance.

## Materials and methods

### Data acquisition in GEO and public-access databases

We downloaded and used processed data from the GSE171023, and GSE231835 datasets deposited in the GEO (https://www.ncbi.nlm.nih.gov/geo/). The GSE171023 dataset contains m(6)A-sequencing data for bladder cancer T24 cells with IGF2BP3 knockout vs. control. The GSE231835 dataset contains RNA-sequencing data for bladder cancer T24 cells with cisplatin resistance vs. control. The public-access database, UALCAN (https://ualcan.path.uab.edu/index.html), was searched to show candidate gene expression based on the TCGA samples of bladder cancer. The RBP-Target analysis using the ENCORI platform (https://rnasysu.com/encori/index.php) and the m6A target database (http://m6a2target.canceromics.org/) were searched to predict the possible sites of m6A modification of SRD5A3.

### Human tissue specimen selection

Primary tumor samples and adjacent normal samples from 30 bladder cancer patients who had undergone radical or partial cystectomy were collected for clinical validation. None of included patients received chemotherapy or radiotherapy before surgical treatment. Among these 30 bladder cancer patients, there were 21 males and 9 females. Based on tumor node metastasis (TNM) staging system detailed in the American Joint Committee on Cancer [28], 30 bladder cancer patients consisted of 22 with stage I or stage II and 8 with stage III, 26 being well differentiated and 4 being poorly differentiated, and 2 with lymph-node metastasis and 28 without. Human tissue specimen selection was performed with the approval of the Ethics Committee of Guangzhou Institute of Cancer Research, the Affiliated Cancer Hospital, Guangzhou Medical University (No. 2024-ZN032).

### Cell culture and lentiviral transduction

Human bladder cancer cell lines, T24 and 5637 (ATCC, Shanghai, China), were cultured in the RPMI-1640 medium (Gibco, Gaithersburg, MD, USA) with the addition of 10% fetal bovine serum (FBS, Gibco). Normal human uroepithelial cell line, SV-HUC-1 (Cell Bank of Type Culture Collection, Shanghai, China), was cultured in the DMEM (Invitrogen, Carlsbad, CA, USA) with the addition of 10% FBS (Gibco). All cells underwent incubation in a humidified atmosphere containing 5%CO_2_ and 95% at 37 °C. Parental T24 and 5637 cells were exposed to gradually increasing doses of cisplatin over the course of 6 months to develop cisplatin-induced drug-resistant bladder cancer cell lines, namely T24R and 5637R cells. At the beginning, T24 and 5637 cells were exposed to a sub-lethal dose of cisplatin which was set just below the level inducing significant cell death while ensuring the survival of a small population of less sensitive cells. To generate the stable cell line, SRD5A3 knockdown (KD) virus, IGF2BP3 KD virus, IGF2BP3 overexpression (OE) virus, and their counterpart control virus were purchased from Shanghai GenePharma Co., Ltd. (Shanghai, China). The lentiviral transduction was preformed based on the manufacturer’s instructions. Supernatants of 293 T cells infected by these lentiviruses were harvested 48 h later followed by purification using ultracentrifugation. T24R and 5637R cells were transduced with the recombinant lentivirus plus 5 μg/ml of Polybrene. A stable transfected strain was selected by a medium with 4 μg/ml puromycin for two generations.

### Quantitative real-time PCR (qRT-PCR)

Extraction of total RNA from human tumor tissue and bladder cancer cells and the following reverse transcription into cDNA were completed by using Trizol reagent (Invitrogen) and PrimeScript RT reagent (Takara, Tokyo, Japan), respectively. The mRNA expression levels of interest genes were determined using SYBR Premix Ex Taq (Takara) with the aid of a Bio-Rad CFX96 PCR system (Bio-Rad, Hercules, CA, USA). The resulting mRNA expression levels of interest genes were normalized GAPDH mRNA and quantification of their relative expressions was completed by the 2^−∆∆Ct^ method. Used primer sequences are: 5’-TTTAATCAGGCCCTGTCTGC-3’ (forward) and 5’-GGGGTATAGAAATGGAATGGAGA-3’ (reverse) for SRD5A3, 5’-TCGTGACCAGACACCTGATGAG-3’ (forward) and 5’-GGTGCTGCTTTACCTGAGTCAG-3’ (reverse) for IGF2BP3, 5’- GGAGCGAGATCCCTCCAAAAT-3’ (forward) and 5’-GGCTGTTGTCATACTTCTCATGG-3’ (reverse) for GAPDH.

### mRNA stability assay

T24R cells and 5637R cells (control and IGF2BP3 knockdown) were seeded into 6-well plates and then treated with 5 µM actinomycin D (Sigma, Missouri, USA) for indicated time points (0, 2, 4, 6, 8, and 10 h), followed by qRT-PCR. The level of remaining mRNA at 2, 4, 6, 8, and 10 h was normalized to the level at 0 h. The one-phase exponential decay curves (from 0 to 10 h) were plotted to reflect mRNA decay kinetics.

### Immunoblotting analysis

The human tumor tissue homogenates and bladder cancer cells reacted in the RIPA lysis buffer to obtain total protein, followed by sodium dodecyl sulfate–polyacrylamide gel electrophoresis (10%) separation, transfer onto polyvinylidene fluoride membrane, and blocking buffer treatment (5% skim milk in TBST). The membranes were incubated with rabbit polyclonal anti-SRD5A3 antibody (PA5-55480, Invitrogen, Carlsbad, CA, USA), mouse monoclonal anti-IGF2BP3 antibody (MA5-27480, Invitrogen), and mouse monoclonal anti-GAPDH antibody (MA5-15738, Invitrogen), followed by rinse with TBST and incubation with secondary antibodies. The signal of immunoblots was visualized using an ECL chromogenic kit (Beyotime, Shanghai, China) and GAPDH normalized the density of each immunoblot. Densitometry analysis of immunoblots was carried out with the aid of the ImageJ software program (NIH, Bethesda, MA).

### CCK-8 assay

T24R and 5637R cells were seeded in 96-well plates (5000 cells in 100 μL per well) in a complete medium and each well was added with 100 μL CCK-8 solution (10% of the total volume) (10 µL, Transgen, Beijing, China) for 2 h incubation. The absorbance at 450 nm was captured. Then, the medium was replaced by complete medium supplemented with 5 μg/ml CDDP, and the plates were incubated for 24 h 48, and 72 h. After each interval, each well was added with CCK-8 solution for 2 h incubation, with the absorbance at 450 nm read.

### Colony formation assay

T24R and 5637R cells were seeded in 6-well plates (500 cells per well) and treated with 5 μg/ml CDDP for 24 h. Then, the cells were maintained in DMEM containing 10% FBS for 2 weeks. The medium was refreshed every two days. After fixing with 4% paraformaldehyde (24 ℃ for 1 h) and staining with 0.1% crystal violet (30 min), colonies with more than 50 cells were imaged and counted for statistical analysis.

### EdU staining

The BeyoClick™ EdU Cell Proliferation Kit (Beyotime) was employed to further examine cell proliferation. T24R and 5637R cells were seeded in 96-well plates (1000 cells per well) and treated with 5 μg/ml CDDP for 24 h. Each well was added with 150 μL EdU solution. After 2 h, each well was added with 4% paraformaldehyde for 30 min. Following 5 min permeabilization, the cells were treated with 50 μL EdU-Click reaction mix for 30 min. The cell nuclei were counterstained with 1 mg/ml HO33342 (Beyotime), and the stained cells were captured under a fluorescence microscope (Nikon, Japan) and imaged.

### Flow cytometry

The Annexin V-FITC/PI Apoptosis Detection Kit (BD Biosciences, San Deigo, CA, USA) with the aid of the BD FACSCalibur flow cytometer (BD Biosciences) was utilized for cell apoptosis assay. T24R and 5637R cells were seeded in 6-well plates (1000 cells per well) and treated with 5 μg/ml CDDP for 24 h. Then, the cells were resuspended in 300 ml of binding buffer followed by the addition of Annexin V-fluorescein isothiocyanate and propidium iodide, 5 μL for each stain.

### Methylated RNA immunoprecipitation-PCR (MeRIP-qPCR)

The Magna MeRIP™ m6A kit (Merck Millipore, MA, USA) was utilized to perform MeRIP procedure. In brief, total RNA from cells were purified by an mRNA Isolation System (Sigma, Missouri, USA), treated by DNase I for 30 min, and made into random fragments (100 nucleotides). Subsequently, the RNA fragments were incubated with a mixture of protein-A/G magnetic beads and m6A antibody (MA5-33030, Invitrogen) or IgG (MA5-42729, Invitrogen). The co-precipitated RNAs were separated using the elution buffer followed by the qRT-PCR procedures to determine the m6A methylation level of SRD5A3.

### RNA immunoprecipitation (RIP) assay

The Magna RIP RNA-Binding Protein Immunoprecipitation Kit (Millipore) was utilized to perform RIP assay of IGF2BP3 and SRD5A3. After treating with a complete RIP lysis buffer (Millipore) in the presence of protease inhibitors and RNase, cell lysates were incubated with either anti-IGF2BP3 antibody (712139, Invitrogen) or IgG (MA5-42729, Invitrogen) and immunoprecipitated with magnetic Protein A/G beads. After proteinase k treatment, the immunoprecipitated RNA and total RNA from the whole cell lysates (input controls) were separated using the elution buffer followed by the qRT-PCR procedures to determine the mRNA enrichment of SRD5A3.

### m6A mutation and luciferase reporter assay

The SRD5A3 3ʹUTR fragments which contained intact m6A sites and mutant m6A motifs (mutant 1, 2, 3, and 1 + 2 + 3), were inserted into the pGL3-control vectors (Promega, Madison, WI, USA). Marked adenosine (A) was replaced by cytosine (C) to generate mutant m6A motifs. For dual-luciferase reporter assays, 100 ng wild-type or mutant SRD5A3 fragments, 200 ng shRNA against IGF2BP3 or scramble shRNA, and 20 ng pRL-TK (Renilla luciferase control reporter vector) were co-transfected into HEK-293 T cells in 24-well plates and incubated for 24–36 h. The Dual-Glo Luciferase system (Promega) was employed to examine the luciferase activity using and the pRL-TK activity normalized the results.

### Animal experiments

Forty Male BALB/c 5–6 weeks nude mice (Charles River, Beijing, China) were implanted subcutaneously with 1 × 10^6^ transfected T24R cells (NC, SRD5A3-KD, NC, and IGF2BP3-KD) or untransfected T24R cells coated with Matrix (Becton Dickinson, Bedford, MA, USA). Once the average volume of subcutaneous tumors had grown to be approximately 100 mm^3^, the mice implanted subcutaneously with untransfected T24R cells were injected with recombinant lentivirus (NC, SRD5A3-KD, NC, and IGF2BP3-KD) via the tail vein [the final lentiviral vector titers were determined to contain 3 × 10^8^ TU per mouse, followed by intraperitoneal injections of CDDP (2 mg/kg) every 3 days for 35 days. On day 35, the mice were euthanized by exposure to prolonged inhalatory anesthesia, and their xenografted tumors were excised, photographed, and measured for weight. All procedures involving experimental animals were approved by Guangzhou Medical University (No. GY2024-572). Animals were treated in strict accordance with the Guidelines for the Care and Use of Laboratory Animals (Eighth Edition) [29]. Significant efforts were done to minimize both animal suffering and the numbers used.

### Statistical analysis

Six independent experiments yielded results presented as mean with standard deviation (s.d.). All statistical analyses involving Student’s *t* test, one-way analysis of variance (ANOVA) plus Tukey’s post hoc test, and repeated-measures ANOVA plus Sidak’s multiple comparisons test were performed with the aid of GraphPad Prism version 8.0 (GraphPad Software, La Jolla, CA, USA) for Windows. A value of *P* < 0.05 was regarded statistically significant.

## Results

### Characterization of SRD5A3 in bladder cancer

**Fig. 1:**
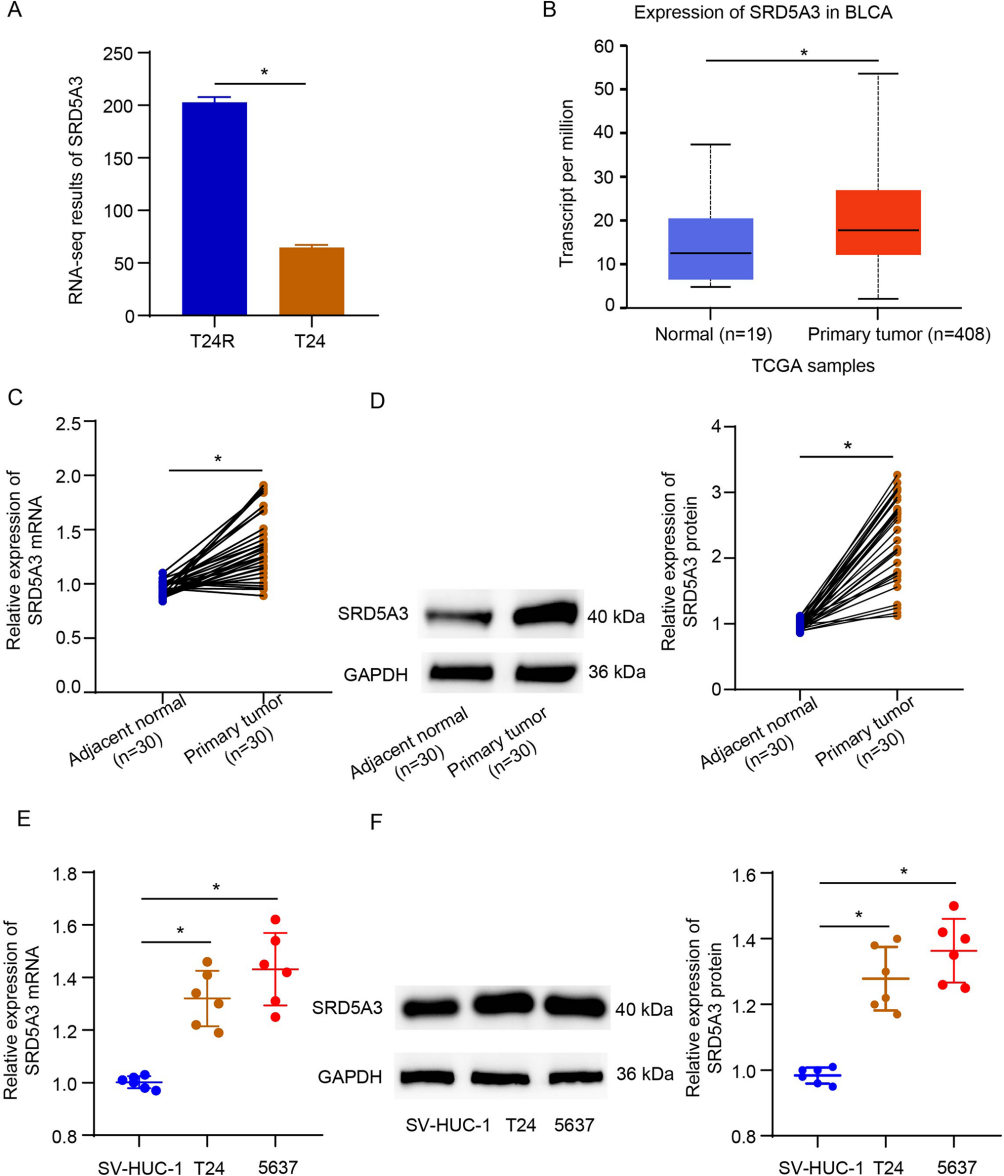
Characterization of SRD5A3 in bladder cancer. **A** SRD5A3 stood out as one of genes upregulated in CDDP-resistant T24 cells compared to parental T24 cells by analyzing the GSE231835 dataset. **B** The UALCAN database showing an increased transcript level of SRD5A3 in the primary bladder urothelial carcinoma (*n* = 408) vs. the normal samples (*n* = 19). **C** and **D** SRD5A3 mRNA and protein expression levels were determined by qRT-PCR and immunoblotting analysis in the corresponding adjacent non-tumor bladder tissues (*n* = 30) and the bladder cancer tissues (*n* = 30). **E** and **F** SRD5A3 mRNA and protein expression levels were determined by qRT-PCR and immunoblotting analysis in standard SV-HUC-1 cells and bladder cancer cells (T24 and 5637). Six independent experiments yielded results. The Student’s *t* tests were performed in panels (**A**–**D**) and the ANOVA plus Tukey’s post hoc test in panels (**E**–**F**). *P < 0.05

First, the GSE231835 dataset was analyzed to identify differentially expressed genes in CDDP-resistant T24 cells compared to parental T24 cells by using the GEO2R bioinformatics tool 23193258. SRD5A3 stood out as one of genes upregulated in CDDP-resistant T24 cells compared to parental T24 cells, with log2FoldChange = 1.41 and *P* < 0.0001 (Fig. 1A), which provoked us to characterize the role of SRD5A3 in the context of bladder cancer. Furthermore, the UALCAN database showed that the transcript level of SRD5A3 was higher in the primary bladder urothelial carcinoma (*n* = 408) than that in the normal samples (*n* = 19) (*P* = 0.0001, Fig. 1B). For clinical validation of SRD5A3 in bladder cancer, we performed qRT-PCR and immunoblotting analysis in these tissue samples to verify immunohistochemical staining results again. Results showed that SRD5A3 exhibited a higher expression at the mRNA level in the tumor tissues of 30 bladder cancer patients than the matched adjacent non-tumor bladder tissues (Fig. 1C), so did at the protein level (Fig. 1D). It was also showed SRD5A3 was lowly expressed at the mRNA and protein levels in standard SV-HUC-1 cells compared to bladder cancer cells (T24 and 5637) (Fig. 1E, F). These data suggest that SRD5A3 may be an oncogene in bladder cancer.

### SRD5A3 knockdown overcomes CDDP resistance in bladder cancer in vitro and in vivo

**Fig. 2:**
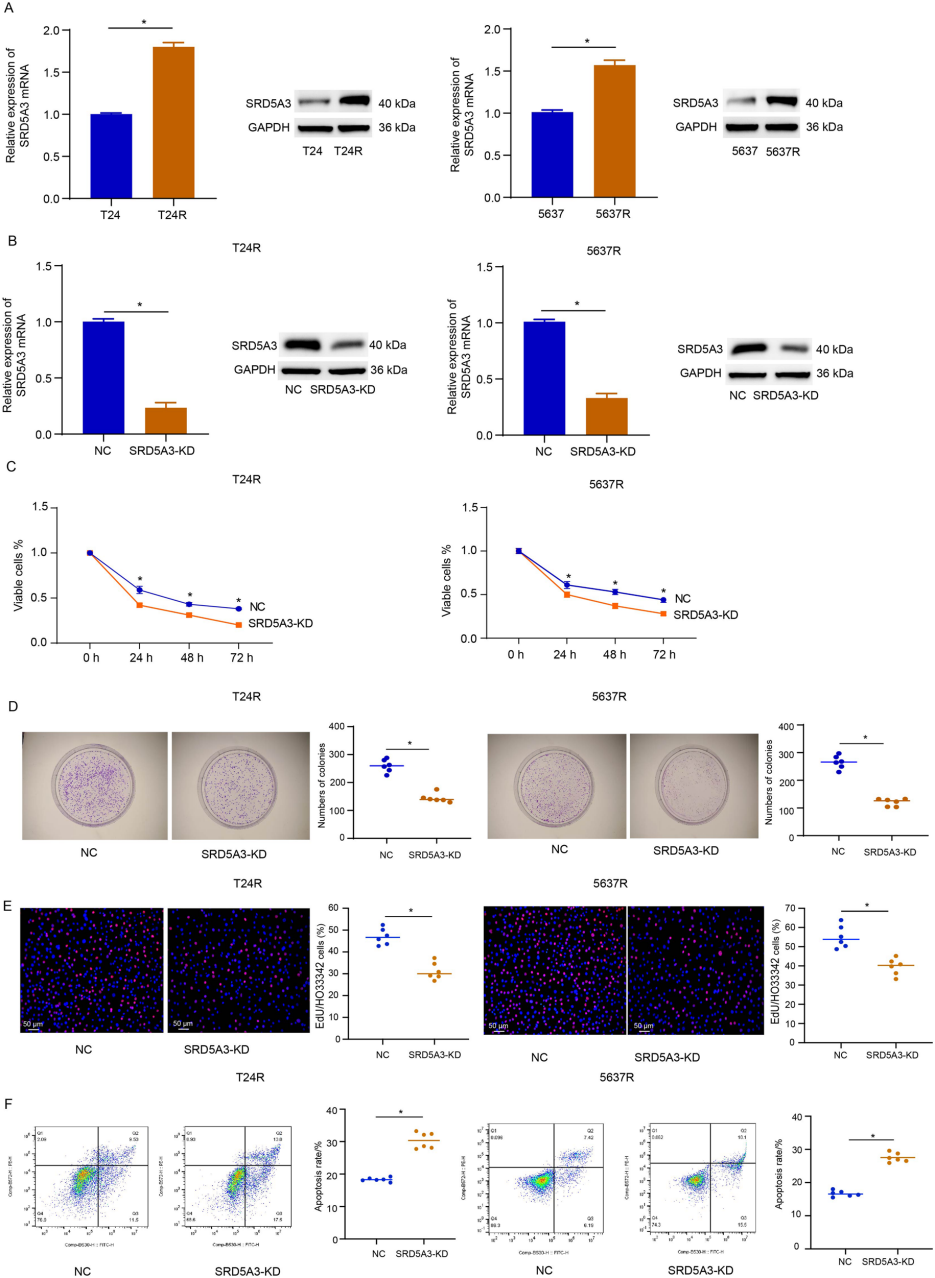
SRD5A3 knockdown overcomes CDDP resistance in bladder cancer in vitro. **A**, **B** SRD5A3 mRNA and protein expression levels were determined by qRT-PCR and immunoblotting analysis in parental cells and CDDP-resistant cells, and CDDP-resistant cells with SRD5A3 knockdown. **C** CCK-8 assay for cell viability of T24R and 5637R cells treated with 5 μg/ml CDDP for 72 h following SRD5A3 knockdown. **D** Colony formation assay for cell proliferation of T24R and 5637R cells treated with 5 μg/ml CDDP for 24 h following SRD5A3 knockdown. **E** EdU staining for cell proliferation of T24R and 5637R cells treated with 5 μg/ml CDDP for 24 h following SRD5A3 knockdown. EdU-positive stained cells were indicated by red spots, and the nuclei were indicated by blue spots. **F** Flow cytometric analysis for cell apoptosis of T24R and 5637R cells treated with 5 μg/ml CDDP for 24 h following SRD5A3 knockdown. Six independent experiments yielded results. The repeated-measures ANOVA plus Sidak’s multiple comparisons test was performed in panel (**C**) and unpaired *t* tests were performed in other panels. *P < 0.05

**Fig. 3:**
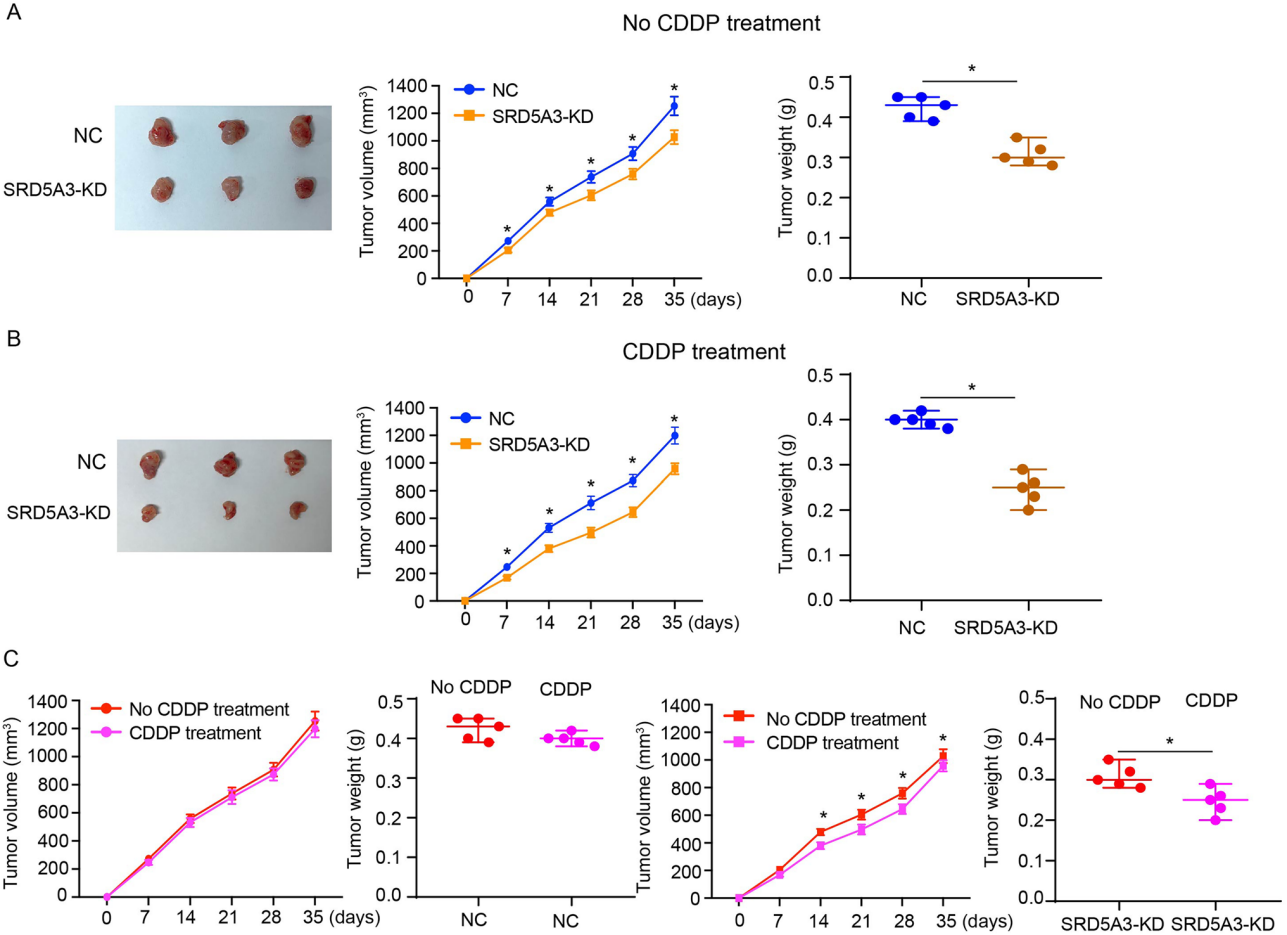
SRD5A3 knockdown overcomes CDDP resistance in bladder cancer in vivo. **A** Representative images of subcutaneous xenograft tumors formed by SRD5A3-knockdown T24R cells for 35 days, and their tumor volume and tumor weight (*n* = 5 for each group). **B** Images of subcutaneous xenograft tumors formed by T24R cells following injections of lentivirus harboring SRD5A3 shRNA and CDDP (2 mg/kg) for 35 days, and their tumor volume and tumor weight (*n* = 5 for each group). **C** The tumor volume and tumor weight of xenograft tumors formed by T24R cells with or without CDDP treatment. The tumor volume was analyzed by repeated-measures ANOVA plus Sidak’s multiple comparisons test. The tumor weight was analyzed by unpaired *t* test. *P < 0.05

As analyzing results of the GSE231835 dataset showing upregulated SRD5A3 in CDDP-resistant T24 cells compared to parental T24 cells, we sought to characterize the relationship between SRD5A3 and CDDP resistance in bladder cancer. T24R and 5637R cells we established exhibited higher expressions of SRD5A3 at the mRNA and protein levels compared to their parental cells (Fig. 2A). SRD5A3 knockdown was achieved in T24R and 5637R cells, as demonstrated by results of qRT-PCR and immunoblotting analysis (Fig. 2B). Results of CCK-8 assay, colony formation assay, and EdU staining revealed that SRD5A3 knockdown reduced cell proliferation and prevented chemoresistance in T24R and 5637R cells treated with CDDP (Fig. 2C–E). Results of flow cytometric analysis revealed that SRD5A3 knockdown led to a pronounced increase in cell apoptosis (Fig. 2F). To determine whether SRD5A3 has the ability to affect the killing effect of CDDP on bladder cancer cells in vivo, we injected transfected or untransfected T24R cells subcutaneously into mice to construct subcutaneous implantation model. Results showed that SRD5A3 knockdown could effectively inhibited the tumor growth of xenografts generated from T24R cells with or without CDDP treatment (Fig. 3A, B). Further analysis found that lentiviral-mediated SRD5A3 knockdown could overcome CDDP resistance in xenografts generated from T24R cells, as demonstrated by significantly reduced tumor growth in CDDP-treated xenografts than those without CDDP treatment (Fig. 3C). These data suggest that SRD5A3 knockdown prevent CDDP resistance in bladder cancer.

### IGF2BP3 directly interacts with SRD5A3 mRNA and increases its stability

**Fig. 4:**
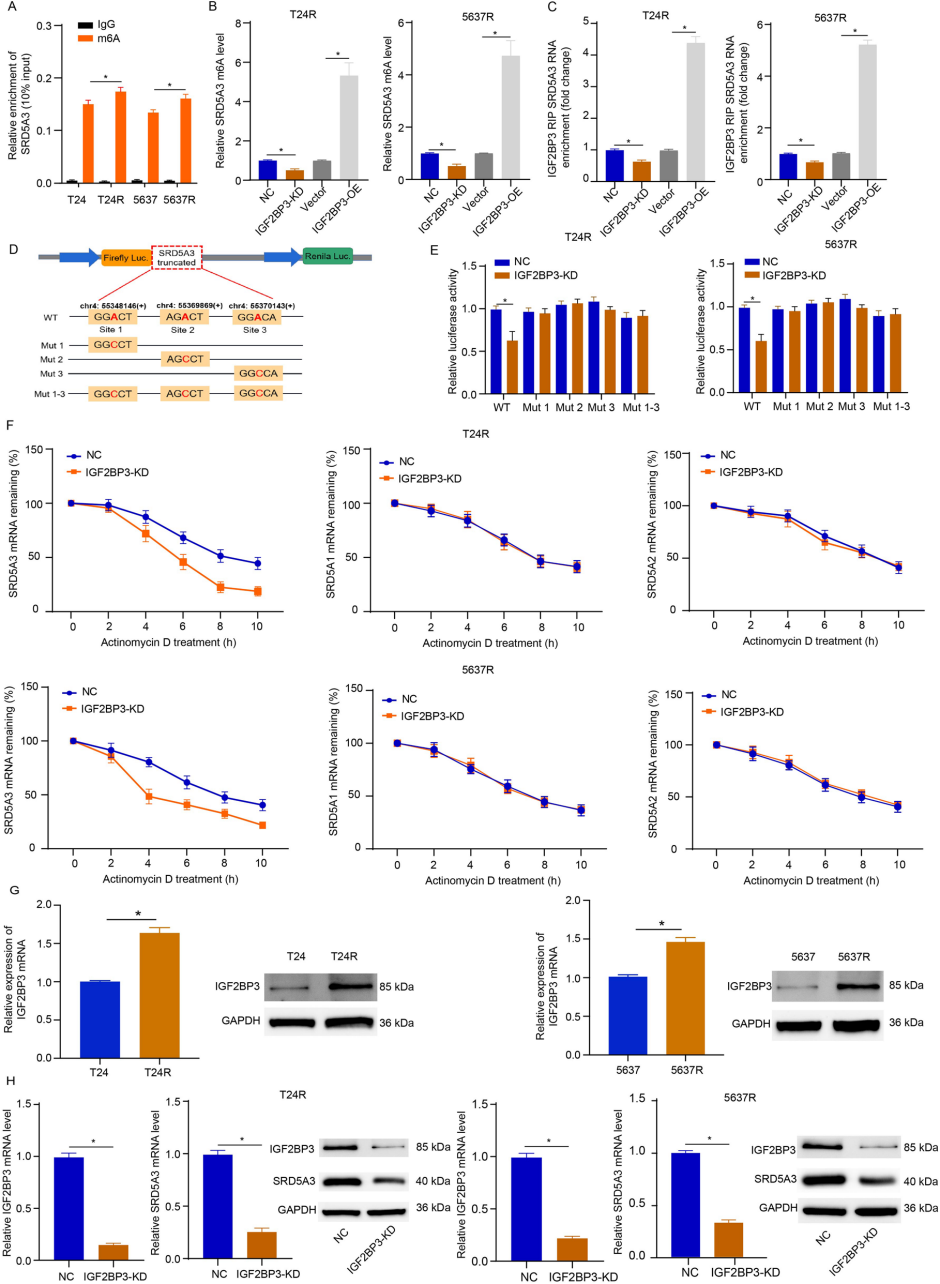
IGF2BP3 directly interacts with SRD5A3 mRNA and increases its stability. **A** The m6A levels of SRD5A3 in T24R and 5637R cells, and their wild-type counterparts were tested by MeRIP-qPCR. **B** The m6A levels of SRD5A3 in T24R and 5637R cells following IGF2BP3 knockdown or overexpression. **C** IGF2BP3 RNP SRD5A3 mRNA enrichment in T24R and 5637R cells following IGF2BP3 knockdown or overexpression. **D** A schematic diagram presenting three methylation sites on SRD5A3 and mutates these sites (**A**–**C**). **E** The luciferase activities of the WT or Mut luciferase reporters in HEK-293 T cells following IGF2BP3 knockdown. **F** The mRNA stability of SRD5A3, SRD5A1, and SRD5A2 in T24R and 5637R cells following IGF2BP3 knockdown after actinomycin D treatment (normalized to 0 h). **G** The IGF2BP3 mRNA and protein expression levels were determined by qRT-PCR and immunoblotting analysis in parental cells and CDDP-resistant cells. **H** SRD5A3 mRNA and protein expression levels were determined by qRT-PCR and immunoblotting analysis in T24R and 5637R cells following IGF2BP3 knockdown or overexpression. Six independent experiments yielded results. The repeated-measures ANOVA plus Sidak’s multiple comparisons test was performed in panel (**F**) and unpaired *t* tests were performed in other panels. *P < 0.05

Next, the IGF2BP3-binding genes were obtained from IGF2BP3 m6A-sequencing data from the GSE171023 dataset and we found that the m6A level of SRD5A3 was significantly reduced in T24 cells with IGF2BP3 knockout relative to their wild-type counterparts (log2FoldChange = − 9.63 and *P* = 0.002), indicating SRD5A3 could be modified by IGF2BP3-mediated m6A modification. Furthermore, we searched the RBP-Target analysis using the ENCORI platform and m6A target database, and predicted results showed that there are multiple m6A motifs that can potentially bind to IGF2BP3 in the case of SRD5A3 mRNA. To examine the regulatory role of m6A modification in SRD5A3 expression during cisplatin resistance development in bladder cancer, we performed MeRIP-qPCR and found an increased m6A levels of SRD5A3 in T24R and 5637R cells compared to their wild-type counterparts (Fig. 4A). A significant decline in m6A-modified SRD5A3 was noted following IGF2BP3 knockdown or overexpression increased the levels of m6a-modified SRD5A3 mRNA in T24R and 5637R cells (Fig. 4B). To verify the role of IGF2BP3 in post-transcriptional modification of SRD5A3 during cisplatin resistance development in bladder cancer, RIP assays revealed enrichment of SRD5A3 in anti-IGF2BP3 antibody fraction, underscoring the interaction between IGF2BP3 and SRD5A3 mRNA. As shown in Fig. 4C, IGF2BP3 knockdown reduced this enrichment in T24R and 5637R cells, while IGF2BP3 overexpression caused a higher enrichment with SRD5A3 mRNA. For better understanding of m6A modification in regulating SRD5A3 expression, we utilized the ENCORI platform to obtain the m6A modification site sequence RRACH (D = A, G or U; R = A or G; H = A, U or C) and constructed a wild-type (WT) and four mutant- (Mut1, 2, 3, 1–3) plasmids to examine the specific modifications of SRD5A3 (Fig. 4D). The wild-type plasmid contained the full-length 3’UTR sequence with intact m6A sites, while each of the mutants made from A to C eliminated the effects of m6A methylation (Fig. 4E). The mRNA stability assays found that IGF2BP3 knockdown resulted in lower mRNA stability owing to the reduced half-life of SRD5A3 transcript following actinomycin D treatment in T24R cells and 5637R cells (Fig. 4F). Considering SRD5A3 as a member of the steroid 5-alpha-reductase family also including SRD5A1 and SRD5A2, we also examined the mRNA stability of SRD5A1 and SRD5A2 in T24R cells and 5637R cells after IGF2BP3 knockdown. It was found that IGF2BP3 knockdown did not cause significant effects on the mRNA stability of SRD5A1 and SRD5A2 in T24R cells and 5637R cells. Of note, we found T24R and 5637R cells with higher expressions of IGF2BP3 at the mRNA and protein levels than their parental cells (Fig. 4G). Additionally, SRD5A3 mRNA and protein expression levels were reduced in response to IGF2BP3 knockdown in T24R and 5637R cells (Fig. 4H). These data suggest that IGF2BP3 directly interacts with SRD5A3 mRNA and increases its stability during cisplatin resistance development in bladder cancer.

### Characterization of IGF2BP3 in bladder cancer

**Fig. 5:**
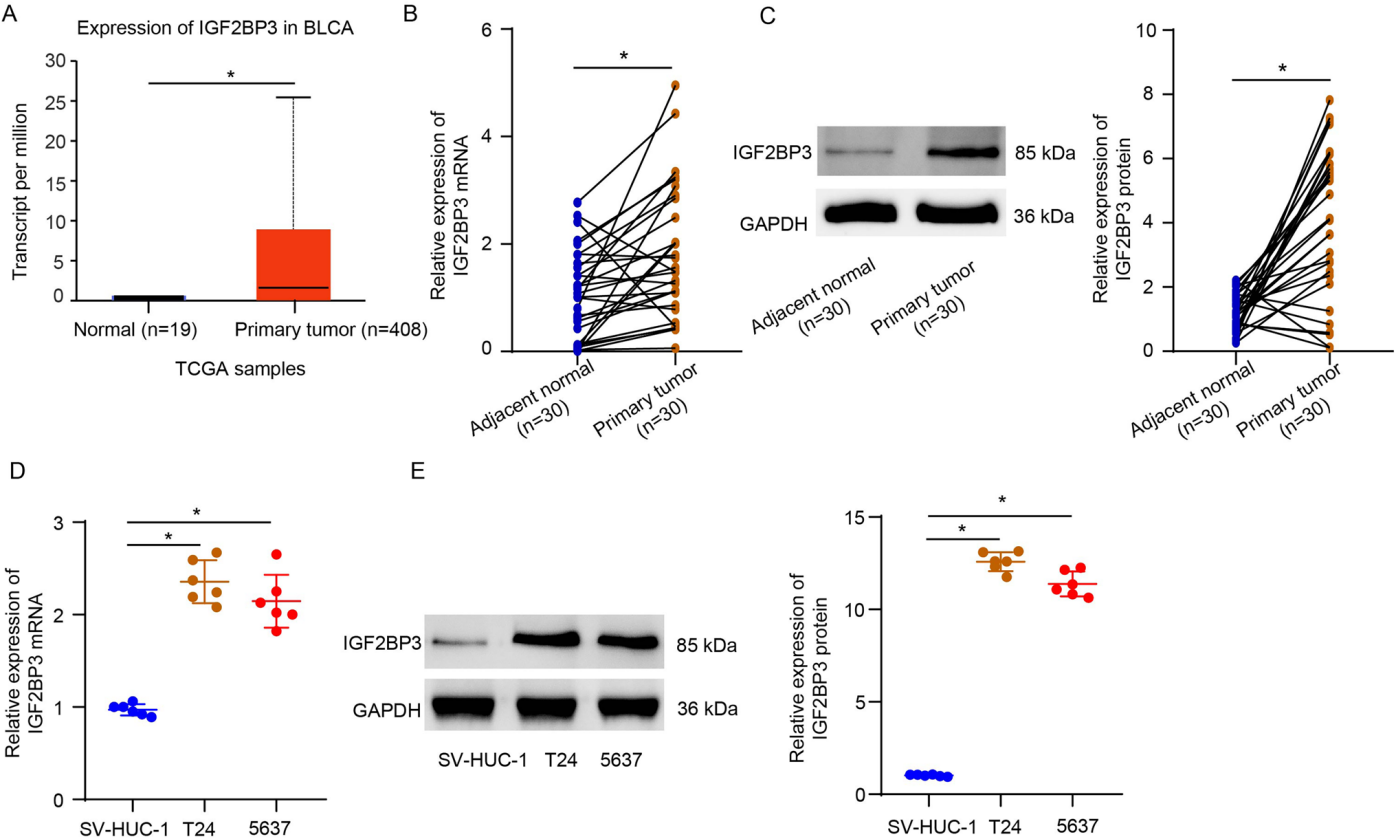
Characterization of IGF2BP3 in bladder cancer. **A** The UALCAN database showing an increased transcript level of IGF2BP3 in the primary bladder urothelial carcinoma (*n* = 408) vs. the normal samples (*n* = 19). **B** and **C** IGF2BP3 mRNA and protein expression levels were determined by qRT-PCR and immunoblotting analysis in the corresponding adjacent non-tumor bladder tissues and the bladder cancer tissues. **D** and **E** IGF2BP3 mRNA and protein expression levels were determined by qRT-PCR and immunoblotting analysis in standard SV-HUC-1 cells and bladder cancer cells (T24 and 5637). Six independent experiments yielded results. The Student’s *t* tests were performed in panels (**A**–**C**) and the ANOVA plus Tukey’s post hoc test in panel (**D**–**E**). *P < 0.05

The UALCAN database showed that the transcript level of IGF2BP3 was higher in the primary bladder urothelial carcinoma (*n* = 408) than that in the normal samples (*n* = 19) (*P* = 0.0001, Fig. 5A). Results of qRT-PCR and immunoblotting analysis displayed elevated mRNA and protein levels of IGF2BP3 in the tumor tissues of 30 bladder cancer patients than the matched adjacent non-tumor bladder tissues (Fig. 5B, C). It was also showed that IGF2BP3 was lowly expressed at the mRNA and protein levels in standard SV-HUC-1 cells compared to bladder cancer cells (T24 and 5637) (Fig. 5D, E). These data suggest that IGF2BP3 may be an oncogene in bladder cancer.

### IGF2BP3 knockdown overcomes CDDP resistance in bladder cancer in vitro and in vivo

**Fig. 6:**
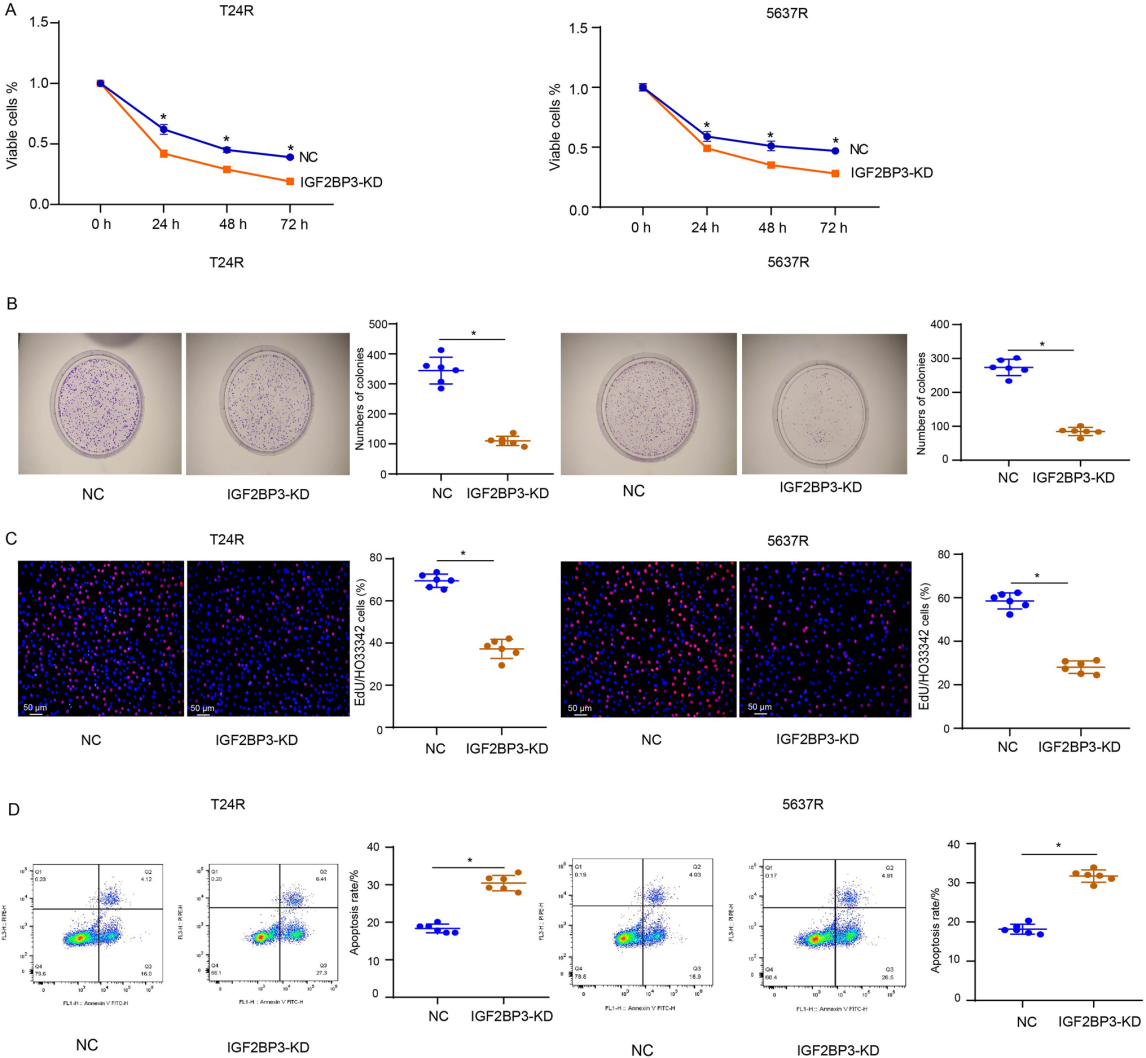
IGF2BP3 knockdown overcomes CDDP resistance in bladder cancer in vitro. **A** CCK-8 assay for cell viability of T24R and 5637R cells treated with 5 μg/ml CDDP for 72 h following IGF2BP3 knockdown. **B** Colony formation assay for cell proliferation of T24R and 5637R cells treated with 5 μg/ml CDDP for 24 h following IGF2BP3 knockdown. **C** EdU staining for cell proliferation of T24R and 5637R cells treated with 5 μg/ml CDDP for 24 h following IGF2BP3 knockdown. EdU-positive stained cells were indicated by red spots, and the nuclei were indicated by blue spots. **D** Flow cytometric analysis for cell apoptosis of T24R and 5637R cells treated with 5 μg/ml CDDP for 24 h following IGF2BP3 knockdown. Six independent experiments yielded results. The repeated-measures ANOVA plus Sidak’s multiple comparisons test was performed in panel (**A**) and unpaired *t* tests were performed in other panels. *P < 0.05

**Fig. 7:**
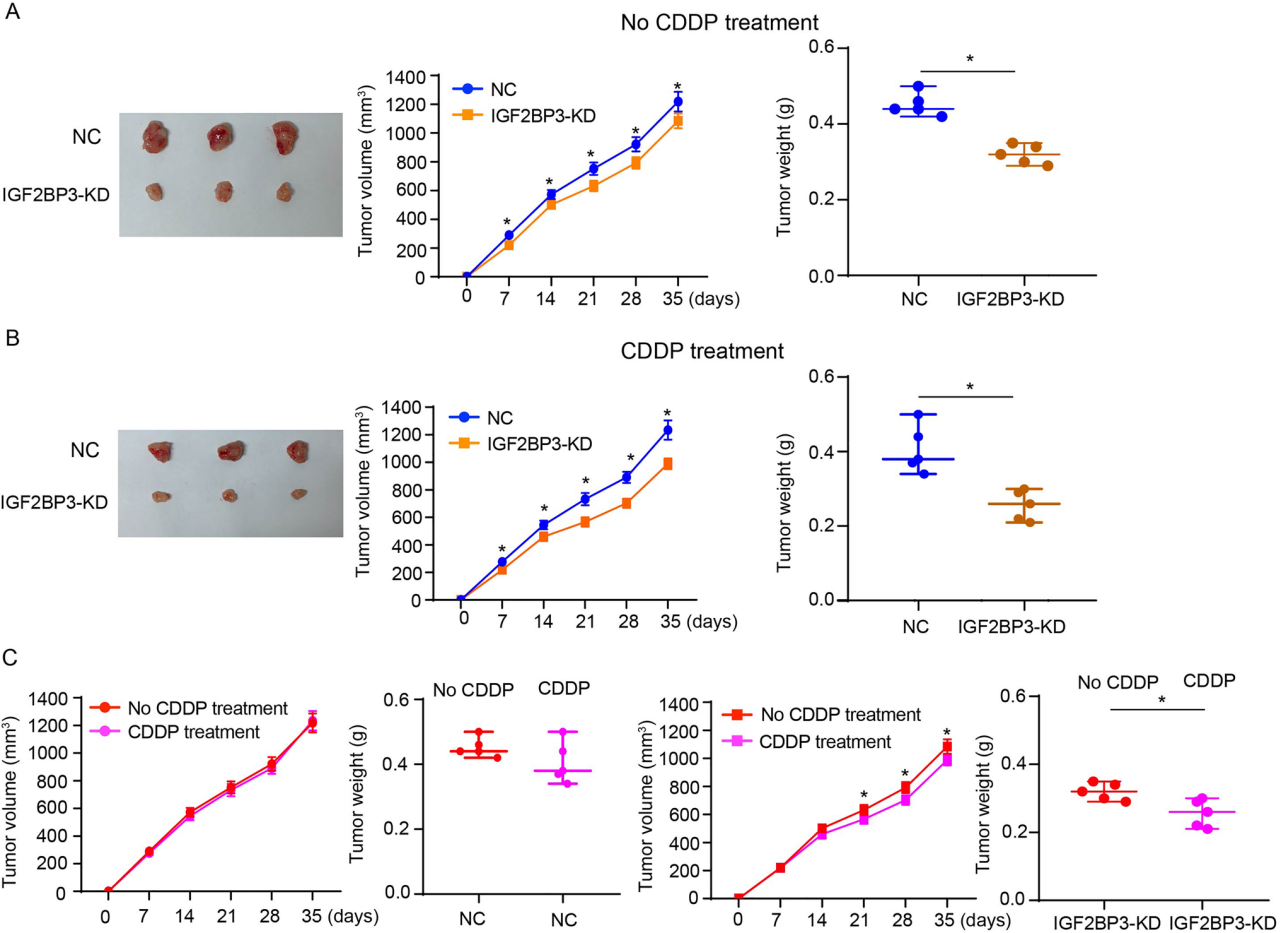
IGF2BP3 knockdown overcomes CDDP resistance in bladder cancer in vivo. **A** Representative images of subcutaneous xenograft tumors formed by IGF2BP3-knockdown T24R cells for 35 days, and their tumor volume and tumor weight (*n* = 5 for each group). **B** Images of subcutaneous xenograft tumors formed by T24R cells following injections of lentivirus harboring IGF2BP3-shRNA and CDDP (2 mg/kg) for 35 days, and their tumor volume and tumor weight (*n* = 5 for each group). **C** The tumor volume and tumor weight of xenograft tumors formed by T24R cells with or without CDDP treatment. The tumor volume was analyzed by repeated-measures ANOVA plus Sidak’s multiple comparisons test. The tumor weight was analyzed by unpaired *t* test. *P < 0.05

Next, we intended to characterize the role of IGF2BP3 during cisplatin resistance development in bladder cancer. Results of CCK-8 assay, colony formation assay, and EdU staining revealed that IGF2BP3 knockdown reduced cell proliferation and prevented chemoresistance in T24R and 5637R cells treated with CDDP (Fig. 6A–C). Results of flow cytometric analysis revealed that IGF2BP3 knockdown led to a pronounced increase in cell apoptosis (Fig. 6D). Animal experiments showed that IGF2BP3 knockdown could effectively inhibited the tumor growth of xenografts generated from T24R cells with or without CDDP treatment (Fig. 7A, B). Further analysis found lentiviral-mediated IGF2BP3 knockdown could overcome CDDP resistance in xenografts generated from T24R cells, as demonstrated by significantly reduced tumor growth in CDDP-treated xenografts than those without CDDP treatment (Fig. 7C). These data suggest that IGF2BP3 knockdown prevents CDDP resistance in bladder cancer.

## Discussion

In this study, we investigated the role of SRD5A3 and IGF2BP3 during bladder tumorigenesis and tumor cells resistant to CDDP. Notably, SRD5A3 and IGF2BP3 were highly expressed in bladder cancer tissues and cell lines. The following cellular and animal studies demonstrated that SRD5A3 and IGF2BP3 knockdown effectively reduced bladder cancer cell proliferation, prevented chemoresistance, and induced cell apoptosis. More importantly, IGF2BP3 recognized the SRD5A3 m6A modification and stabilized its mRNA. Therefore, we concluded that SRD5A3 and IGF2BP3 play oncogenic roles in bladder tumorigenesis, and IGF2BP3 directly interacts with SRD5A3 mRNA and increases its stability during cisplatin resistance development in bladder cancer.

SRD5A3 belongs to steroid 5-alpha-reductase family which is required for the generation of steroid hormones, testosterone conversion to DHT, and male sexual development [30]. Steroid hormone-mediated signaling pathways have implicated in urothelial tumorigenesis, including bladder cancer [31]. The activation of sex steroid hormone-mediated signaling could affect bladder cancer cell sensitivity to conventional non-surgical therapy [32]. Earlier studies demonstrated that more production of DHT markedly increased bladder cancer cell proliferation and invasion [33]. DHT, as an androgen, may function by binding with nuclear androgen receptor (AR) to promote the development of bladder cancer [34]. The activation of AR signaling has been reported to induce resistance to CDDP treatment [35]. The disparities in androgen DHT levels between men and women may explain the higher incidence rate of bladder cancer in men than women. Thus, DHT-mediated the activation of AR signaling is considered as an attractive target for the management of bladder cancer in male patients [36]. Among a high portion of male patients with bladder cancer and expressing AR, the combination of AR inhibitor, Enzalutamide, and CDDP synergistically inhibited bladder cancer cell growth more effectively than single agent alone [37]. SRD5A3 was found with a low expression level in women than man [38]. A Korean study found that the short AC repeats of SRD5A3 polymorphism may confer a genetic predisposition to prostate cancer [39]. AR is recruited to a negative androgen response element on the promoter of SRD5A3 and the different expression levels of 5α-reductase isoenzymes affect patient response to 5α-reductase inhibitors in prostate cancer [40]. Our results revealed an upregulation of SRD5A3 in bladder cancer tissues and SRD5A3 knockdown could reduce bladder cancer cell proliferation, prevent CDDP resistance, induce cell apoptosis, and inhibit bladder tumorigenesis in mice.

In this study, we revealed the regulation of SRD5A3 mRNA expression mediated by m6A reader IGF2BP3 during cisplatin resistance development in bladder cancer. SRD5A3 as one of the IGF2BP3-binding genes were noted by analyzing the GSE171023 dataset which contains m6A-sequencing data in T24 cells with IGF2BP3 knockout relative to their wild-type counterparts. Our further results obtained from MeRIP-qPCR showed an increased m6A levels of SRD5A3 in T24R and 5637R cells compared to their wild-type counterparts. Although no previous evidence showed the regulation of SRD5A3 by epigenetic modifications in cancers, SRD5A2 methylation in the promoter region leading to low expression was indicative of better survival for castration-resistant prostate cancer patients after androgen deprivation therapy [41], indicating the regulation of 5-alpha-reductase isoforms by epigenetic modifications in prostate cancer. The m6A modifications have been involved in the transition to chemoresistance in bladder cancer [42]. The m6A clusters and patterns were found to modulate tumor immunity, providing novel comprehensive strategies in treating bladder cancer [43]. The expression of IGF2BP3 is related to disease recurrence and cancer-specific mortality in urothelial carcinoma, which could improve risk stratification of urothelial carcinoma patients undergoing radical nephroureterectomy [44]. IGF2BP3-mediated mRNA stability of multiple oncogenes, such as CDK2 and STAT3 via a m6A-dependent manner, have been found to promote the development of bladder cancer [45, 46]. Our results revealed an upregulation of IGF2BP3 in bladder cancer tissues and IGF2BP3 knockdown could reduce bladder cancer cell proliferation, prevent CDDP resistance, induce cell apoptosis, and inhibit bladder tumorigenesis in mice.

In conclusion, the study provides compelling in vitro and in vivo evidences proving the m6A modification machinery between SRD5A3 and IGF2BP3 in bladder cancer. IGF2BP3-mediated SRD5A3 m6A modification induces acquired CDDP resistance in bladder cancer cells. Our findings provide a better knowledge of the epitranscriptional regulation mechanisms of SRD5A3 expression and the biological and epigenetic importance of m6A reader IGF2BP3 in bladder tumorigenesis. Therefore, the combined treatment with CDDP and specific inhibitors for IGF2BP3 or m6A, can be considered in a clinical setting to overcome intrinsic or acquired resistance to CDDP in bladder cancer patients. However, we must admit the mechanism by which SRD5A3 contributes to cisplatin resistance is unclear in this study, which will be the most important issue needed to be addressed in further studies.

## Data Availability

Data are provided within the manuscript or supplementary information files.
